# Expression of Endothelial NOX5 Alters the Integrity of the Blood-Brain Barrier and Causes Loss of Memory in Aging Mice

**DOI:** 10.3390/antiox10081311

**Published:** 2021-08-20

**Authors:** Adriana Cortés, Maite Solas, Álvaro Pejenaute, Miguel A. Abellanas, Marcos Garcia-Lacarte, Maria S. Aymerich, Javier Marqués, María J. Ramírez, Guillermo Zalba

**Affiliations:** 1 Department of Biochemistry and Genetics, University of Navarra, 31008 Pamplona, Spain; acortes.3@alumni.unav.es (A.C.); apejenaute@alumni.unav.es (Á.P.); mabellanas.1@alumni.unav.es (M.A.A.); mglacarte@unav.es (M.G.-L.); maymerich@unav.es (M.S.A.); jmarquesc@unav.es (J.M.); 2 IdiSNA, Instituto de Investigación Sanitaria de Navarra, 31008 Pamplona, Spain; 3 Department of Pharmacology and Toxicology, University of Navarra, 31008 Pamplona, Spain; msolaszu@unav.es (M.S.); mariaja@unav.es (M.J.R.); 4 Neuroscience Program CIMA, University of Navarra, Navarra Institute for Health Research (IdiSNA), 31008 Pamplona, Spain

**Keywords:** oxidative stress, NOX5, aging, occludin, Zonula occludens-1, tight junctions

## Abstract

Blood-Brain barrier (BBB) disruption is a hallmark of central nervous system (CNS) dysfunction, and oxidative stress is one of the molecular mechanisms that may underlie this process. NADPH oxidases (NOX) are involved in oxidative stress-mediated vascular dysfunction and participate in the pathophysiology of its target organs. The NADPH oxidase 5 (NOX5) isoform is absent in rodents, and although little is known about the role it may play in disrupting the BBB, it has recently been implicated in experimental stroke. Our aim was to investigate the role of NADPH oxidase 5 (NOX5) in promoting vascular alterations and to identify its impact on the cognitive status of aged mice. No differences were detected in the arterial blood pressure or body weight between knock-in mice expressing endothelial NOX5 and the control mice. The Morris water maze test showed memory impairments in the aged knock-in mice expressing NOX5 compared with their control littermates. For assessing the BBB integrity, we studied the protein expression of two tight junction (TJ) proteins: Zonula occludens-1 (ZO-1) and occludin. Compared to the control animals, *Aged NOX5* mice exhibited reduced levels of both proteins, demonstrating an alteration of the BBB integrity. Our data indicate that vascular NOX5 may favor behavioral changes with aging through oxidative stress-mediated BBB breakdown.

## 1. Introduction

In 2050, 22% of world’s population will be above 60 years old. People worldwide above this age will increase from 900 million in 2015 to two billion by 2050 [1]. This aging population will result in an eruption of age-related diseases like diabetes mellitus, cardiovascular diseases, cancer or neurodegenerative disorders [2]. Throughout aging, the energy supply decreases, and progressive degeneration together with proinflammatory and senescence processes takes place. In this way, cardiac, muscular and endothelial progenitor senescent cells have been associated with endothelial and cardiac dysfunction, leading to the progression of several diseases, such as hypertension, atherosclerosis, heart failure and even stroke [3]. These endothelial dysfunctions and vascular alterations may induce Blood-Brain barrier (BBB) disruption, a hallmark of central nervous system (CNS) alterations [4].

The BBB is a multicellular vascular structure that separates the CNS from the circulating peripheral blood, constituting the first defense of the brain against external aggressions. It is composed by several cell types, including astrocytes-end-feet and pericytes, but predominantly by endothelial cells attached to each other by adherens and tight junctions (TJ) [5]. TJ, composed by strong protein bonds, are key in BBB permeability prevention. These unions are selectively permeable and are able to discriminate between solutes according to their size and charge [6]. Zonula occludens-1 (ZO-1) and occludin regulate the BBB permeability through interactions between the cytoskeleton and TJ. ZO-1 is a peripheral membrane protein with multiple sites of protein-protein interactions that organizes and regulates the TJ, while occludin is a transmembrane protein that may regulate the structure of TJ [7]. 

BBB dysfunction and breakdown leads to leakages of harmful blood components into the CNS, aberrant molecule transport and clearance, as well as cellular infiltration [8]. Functional BBB integrity can be disrupted by neuroinflammation and oxidative stress, associated with many age-related disorders, such as Alzheimer’s disease [9] or Parkinson’s disease [10]. Reactive oxygen species (ROS) accumulation affects cell signaling and triggers cellular senescence, inflammation, apoptosis and necrosis [11]. The NADPH oxidases (NOX) family constitutes the major enzymatic source of ROS in mammalian vasculature [12]. NOX-derived ROS are produced within the CNS and are related with neuropathology [13,14,15]. For instance, it is described that NOX2 and NOX4 chronic activation is harmful in the brain [16]. Concerning TJ, ROS produced by NOX downregulate occludin, promote its internalization from the membrane and, therefore, reduce its contribution to BBB tightness [17]. Additionally, NOX-derived ROS induce ZO-1 downregulation and, subsequently, BBB disruption [18]. Within the NOX family, NOX5 is the last discovered isoform, studied to a lesser extent because of its evolutive loss in the rodent genome. The biological function of NOX5 is to generate superoxide anion, a known mediator of cerebrovascular damage, following stroke [19]. In an ischemia-reperfusion mouse model, endothelial NOX5 expression produced a higher infarct size after stroke. This effect was promoted via ROS-dependent BBB permeabilization [20]. In the current work, we studied the effect of NOX5 endothelial expression in an aging mouse model. We hypothesized that the chronic endothelial NOX5 expression in aging mice may induce, among other things, BBB integrity disruption, leading to altered cognitive function.

## 2. Materials and Methods

### 2.1. Conditional Knock-In Aging Mice

Conditional C57BL/6 knock-in mice expressed NOX5-β only in endothelial cells after its intraperitoneal injection of tamoxifen (40 mg/kg) on 3 nonconsecutive days (NOX5^+/−^CRE^+/−^). There are many variants of NOX5, but for the knock-in mouse model, the β isoform of the oxidase was chosen, as it is the most expressed in human vasculature [21]. This activated endothelial-specific CRE recombinase, triggering transgenesis [22] (Figure S1A). Two experimental designs were conducted. On the one hand, for NOX5-β knock-in model characterization, NOX5-expressing mice (NOX5^+/−^CRE^+/−^) were used, and wild-type (WT) mice and mice expressing endothelial-specific CRE recombinase were considered as controls (*Cdh5*(PAC)-CreERT2; CRE^+/−^) (gently provided by Wang et al. [23]). On the other hand, 3 groups were created for the aged phenotype study: (i) young (2 months) CRE^+/−^ and NOX5^+/−^CRE^+/−^ genotype mice (*n* = 12, *Control Young* group), (ii) aged (21 months) CRE^+/−^ genotype mice (*n* = 19, *Aged* group) and (iii) aged (21 months) NOX5^+/−^CRE^+/−^ genotype mice (*n* = 13, *Aged NOX5* group). A tamoxifen induction was performed 6-8 weeks after birth, and booster doses were given to the aging mice at months 9, 12 and 19. The aging mice were weighted monthly, and their blood pressure was also analyzed periodically using a noninvasive tail cuff system with a MRBP single-animal tail cuff blood pressure multichannel system (IITC Life Science). The animals were euthanized (at month 21) by decapitation after the behavioral tests were performed, and their organs were extracted and processed accordingly (Figure S1B). Brains from the aging models were removed and dissected on ice to avoid tissue degradation. Then, the hippocampi were also collected and stored at −80 °C. For the immunofluorescence studies of the aging mice, the right hemispheres (*n* = 5) were fixed using 4% paraformaldehyde in 0.1-M phosphate-buffered saline (PBS) (pH = 7.4) for 72 h. Later, the samples were soaked in 30% sucrose solution, and, finally, the brains were cut into a series of 40-μm slides. 

The experiments were performed in accordance with the European Communities Council Directives (2010/63/EU) guidelines for the care and use of laboratory animals and were approved by the University of Navarra Animal Research Review Committee (Protocol 141-15).

### 2.2. Real-Time Quantitative PCR Assay

The total RNA was obtained from the different organs by homogenizing in TRIzol (Thermo Scientific, Rockford, IL, USA) and following the standard methods. The RNA quantity and quality were evaluated by a Nanodrop ND-1000 spectrophotometer (Thermo Scientific, Rockford, IL, USA). Then, 1.5 μg of the total RNA of each sample were reverse-transcribed into cDNA using the SuperScript III cDNA Synthesis Kit (Thermo Scientific, Rockford, IL, USA). Real-time PCRs were performed using the iQ SYBR Green Supermix Kit (Bio-Rad, Hercules, CA, USA) in an CFX384 Touch Real-Time PCR Detection System (Bio-Rad, Hercules, CA, USA). As the housekeeping gene, Glyceraldehyde 3-phosphate dehydrogenase (GAPDH) was chosen. The specific primers used for cDNA amplification are shown in Table 1.

### 2.3. Immunofluorescence (IF)

For the NOX5-β knock-in model characterization, the brain sections were fixed using 4% paraformaldehyde for 10 min, washed (3 × 5 min) with PBS 0.1 M (pH = 7.4) and incubated in blocking solution (PBS containing 10% FBS and 1% 0.3-M glycine) for 1 h at room temperature. The sections were incubated with the primary antibodies overnight at 4 °C and, for 1 h, protected from light at room temperature with the secondary antibodies. The primary antibodies used were anti-NOX5 (1:100, Abcam, Cambridge, MA, USA) and anti-CD31 (1:100, BD Biosciences, San Jose, CA, USA). The secondary antibodies used were Alexa Fluor 488 goat anti-rat and Alexa Fluor 594 goat anti-rabbit (1:500, Invitrogen-Molecular Probes, Eugene, OR, USA). The sections were prepared using Vectashield (Vector Laboratories, Burlingame, CA, USA) mounting medium with DAPI. The microscope Nikon Eclipse 80i (Nikon Instruments Inc, Tokyo, Japan) was used for image obtaining.

In the aging model, the free-floating brain sections were washed 3 times for 10 min each with PBS 0.1 M (pH = 7.4). Then, they were incubated in blocking solution (PBS containing 0.3% Triton X-100, 0.1% BSA and 2% normal donkey serum) for 2  h at room temperature. The sections were incubated with the primary antibodies diluted in the blocking solution overnight at 4 °C. Afterwards, the sections were incubated with the secondary antibodies also diluted in the blocking solution for 2  h protected from light at room temperature. To visualize the brain microvessels, aside from the CD31 marker, fluorescein-conjugated Lycopersicon esculentum lectin (1:200, Vector Laboratories, Burlingame, CA, USA) was used and incubated together with the secondary antibody. The primary antibodies used were anti-fibrin (1:1000, Dako, Santa Clara, CA, USA), anti-nitrotyrosine (1:100, Millipore, Darmstadt, Germany) and anti-CD31 (1:100, BD Biosciences, San Jose, CA, USA). Additionally, the secondary antibodies used were Alexa Fluor 546 goat anti-rabbit (1:400, Invitrogen-Molecular Probes, Eugene, OR, USA), Alexa Fluor 488 goat anti-rat and Alexa Fluor 594 donkey anti-mouse (1:500, Invitrogen-Molecular Probes, Eugene, OR, USA). For the immunoglobulin G (IgG) staining, an incubation of brain slides with only the secondary antibody Alexa Fluor 546 goat anti-mouse (1:1000, Invitrogen-Molecular Probes, Eugene, OR, USA) for 2 h at room temperature was performed. All the sections were processed at the same time under identical conditions to ensure comparable fluorescence signals, which were detected with confocal microscope LSM 510 Meta (Carl Zeiss, Oberkochen, Germany).

The images were randomly taken from three nonadjacent tissue sections per specimen (*n* = 4) and analyzed using NIH-developed ImageJ. To quantify the capillary leakage, the levels of extravascular fibrin and IgG were measured as previously described [24,25,26,27]. Briefly, the ImageJ Area tool was used to measure the total area of fibrin- and IgG-positive signals, and, when it colocalized with the lectin-positive signal, it was subtracted from the total area of leakage. Using this method, a value representing the extravascular levels of each plasma-derived protein was obtained. All the images were analyzed by a blinded investigator. 

### 2.4. Dihydroethidium Staining

Dihydroethidium staining (DHE, Thermo Fisher) was performed on fresh brain sections from the control mice to analyze the intracellular superoxide anion production. Briefly, 10-µm slides were incubated with PBS 0.1 M (pH = 7.4) containing 2-µM DHE for 30 min at 37 °C and then washed three times for 5 min each in PBS. The sections were mounted using Vectashield (Vector Laboratories, Burlingame, CA, USA) mounting medium with DAPI. The microscope Nikon Eclipse 80i (Nikon Instruments Inc, Tokyo, Japan) was used for image obtaining.

### 2.5. Western Blotting

Lung protein extraction was performed with the RIPA Buffer (1% NP-40, 150-mM NaCl, 50-mM Tris, pH = 8, 0.1% SDS, 0.5% sodium deoxycholate and EDTA-free protease inhibitors). The samples were sonicated to ensure membrane degradation. Total hippocampal tissue homogenates were obtained by homogenizing the hippocampus in ice-cold lysis buffer (NaCl 200 mM, HEPES 100 mM, glycerol 10%, NaF 200 mM, Na_4_P_2_O_7_ 2 mM, EDTA 5 mM, EGTA 1 mM, DTT 2 mM, PMSF 0.5 mM, Orthovanadate 1 mM and NP-40, inhibitors of phosphatases and proteases inhibitors at 1%) and centrifuged at 13,000 rpm 4 °C for 20 min. The supernatant was aliquoted and frozen at −80 °C until use. The homogenates (30 μg of protein) were separated by electrophoresis on polyacrylamide gels (5–10%) and transferred into nitrocellulose membranes, which were incubated with the primary antibodies overnight at 4 °C. The primary antibodies used were anti-NOX5 (1:1000, Abcam, Cambridge, MA, USA), anti-ZO-1 (1:1000, Invitrogen-Molecular Probes, Eugene, OR, USA) and anti-Occludin (1:1000, Invitrogen-Molecular Probes, Eugene, OR, USA). The secondary antibodies conjugated to IRDye 800CW or IRDye 680CW (LI-COR Biosciences, Lincoln, NE, USA) were diluted to 1:5000 in TBS with 5% BSA or 5% milk. Finally, the bands were visualized using an Odyssey Infrared Imaging System (LI-COR Biosciences, Lincoln, NE, USA). β-actin (mouse monoclonal, 1:10000, Sigma-Aldrich, St. Louis, MO, USA) or α-tubulin (mouse monoclonal, 1:10,000, Sigma-Aldrich, St. Louis, MO, USA) were used as the internal controls.

### 2.6. Flow Cytometry Assay

Mice expressing NOX5 (*n* = 2, NOX5^+/−^CRE^+/−^) and the negative control (*n* = 1, WT) were used to check if its expression was limited to the endothelium by flow cytometry. Peripheral blood samples were collected in tubes with EDTA, diluted in PBS and treated with ammonia-chloride-potassium (ACK) to ensure erythrocytes lysis. NOX5 expression was analyzed by combined immunofluorescence staining directed against the cell surface and intracytoplasmic proteins in peripheral blood samples from our transgenic mice. First, cells from the peripheral blood were marked 30 min at 4 °C using combinations of extracellular monoclonal antibodies directly conjugated with a fluorophore shown in Table 2, and 7AAD was used as the viability maker.

Cell fixation and permeabilization prior to NOX5 protein staining was performed using the Fixation/Permeabilization Solution Kit (BD Biosciences, San Jose, CA, USA) according to the standard methods. NOX5 was immunodetected using the NOX5 primary rabbit antibody kindly donated by Lambeth et al. [28]. After an incubation of 30 min at 4 °C with the primary antibody (1:100), all the samples were incubated for 30 min with the goat anti-rabbit IgG (H + L) (Alexa Fluor 488, Invitrogen-Molecular Probes, Eugene, OR, USA, A-11034) secondary antibody (1:500). NOX5-β-infected-TeloHAEC (multiplicity of infection 50), obtained as previously described [22], was used as a positive control of NOX5 expression. NOX5-β-infected TeloHAEC and a pool of all the peripheral blood samples incubated only with the secondary anti-rabbit Alexa Fluor 488 antibody were used as negative controls. The acquisition was performed in a CytoFLEX-Beckman Coulter cytometer. To differentiate the blood subpopulations, B cells were defined as B220+, T cells as CD3+, dendritic cells as CD11b+/CD11c+, monocytes as CD11b+/Ly-6C- and macrophages as CD11b+/Ly-6C-/F4/80+. For the data analysis, FlowJoTM v.10 software (Tree Star, Ashland, OR, USA) was used.

### 2.7. Locomotor and Behavioral Tests

#### 2.7.1. Open Field Test

An open field (35 × 35 cm, 45 cm height) made of black wood was used to evaluate the locomotor activity. The experiment was performed for 30 min and measured using a video tracking system (Ethovision 11.5, Noldus Information Technology B.V., Wageningen, The Netherlands) in a softly illuminated room. The total path (cm) was analyzed.

#### 2.7.2. Rotarod Test

Locomotor coordination was measured in this test using a rotarod (LE8205, Panlab, Barcelona, Spain). The mice were placed on top of the already revolving beam with a constant acceleration going from 4 rpm to 40 rpm in 5 min. The time spent by each mouse walking on the rod was recorded for a maximum of 5 min. All the animals were trained with 3 trials. The results were expressed as the average of the latency to fall and were calculated from 2 consecutive trials, with a resting time of 30 min in between them.

#### 2.7.3. Pole Test

In the pole test, the mice were placed head upwards on the top of a vertical and rough-surfaced pole (height 50 cm; diameter 1 cm). All the animals were trained for 3 times until they were able to turn downward and to descend from the pole. The average time spent by mice in turning downward and completely descending the pole was measured in 2 consecutive trials, with a resting time of 30 min in between them.

#### 2.7.4. Coat Hanger Test

Motor coordination was measured in the coat hanger test. The mice were placed in the middle of a steel coat hanger, and they scored 1 point if they could remain on the hanger at least for 30 s and another point if they lifted 1 hindlimb to the hanger, 2 points if they lifted 2 hindlimbs or 3 points if they lifted the 2 hindlimbs and went to one extreme of the hanger. Each animal was scored twice consecutively without training or resting time between the trials.

#### 2.7.5. Elevated Beam Test

The elevated beam test consisted of an elevated platform under a 60-watt light bulb connected to a cage by a 70-cm elevated beam. The mice were trained for 3 times to cross from the light platform to the cage. The movement was recorded, and the average time to cross was measured in 2 consecutive trials, with a resting time of 30 min in between them.

#### 2.7.6. Novel Object Recognition Task (NORT)

This assay was performed in a square divided into 4 sections (35 cm × 35 cm × 45 cm each) with black walls. For the adaptation of the mice to the space, the day before the experiment, the animals were placed in the square for 30 min. During the sample phase, the mice were allowed to explore for 5 min the 2 identical objects placed inside the cubicle. One hour after, the second task was performed, replacing 1 object by another, and the exploration time was recorded for 5 min. The results were obtained using a video tracking system (Ethovision 11.5; Noldus Information Technology B.V., Wageningen, The Netherlands) and expressed as the percentage of time spent exploring the new object with respect to the total exploration time (discrimination index).

#### 2.7.7. Fear Conditioning Test (FC)

To analyze fear memory, the FC test was used. This experiment consists of 3 phases: habituation, training and test. Firstly, during the habituation phase, the mice were exposed for 3 min to the conditioning chamber with no stimuli presented. A day after the adaptation, the training phase was performed, and the mice were placed for 2 min again in the same chamber, but this time, they were allowed to explore. Then, the mice received a foot shock (0.3 mA) of 2 s, followed by another foot shock 30 s later and were returned to their home cages after another 30 s. The following day, the mice were placed again in the conditioning chamber and allowed to explore the environment for 2 min. Freezing behavior was recorded during this time, and the freezing scores were expressed as percentages. It was carried out in the StartFear System (Panlab).

#### 2.7.8. Morris Water Maze (MWM)

The MWM, a hippocampus-dependent learning task, was used to test spatial memory and to evaluate the working and reference memories. The water maze consists of a circular pool (145 cm diameter), which is virtually divided into 4 equal quadrants (northeast, northwest, southeast and southwest) and filled with water (21–22 °C). For the training of the mice, the platform was placed in the northeast quadrant 1 cm below the water surface. This training was performed over 9 consecutive days (4 trials/day). In order to guide the mice to the hidden platform, several large visual cues were placed in the room. Each trial was finished when the mouse reached the platform (escape latency) or after 60 s, whichever came first. In case the mice failed to reach the platform, they were guided onto it. Once the mice reached the platform, they remained on it for 15 s. To whether evaluate the mice’s memory, probe trials were performed on the 4th, 7th and last day of the test (10th day). During the probe trials, the mice were allowed to swim for 60 s in the pool but without the platform. The results were expressed as the percentage of time spent by each mouse in the target quadrant. All the trials were monitored by a video camera set above the center of the pool and connected to a video tracking system (Ethovision 3.0; Noldus Information Technology B.V., Wageningen, Netherlands).

### 2.8. Statistical Analysis

The data showed the mean ± standard error of the mean (SEM). The statistical analysis was performed using GraphPad Prism 8 (GraphPad®, San Diego, CA, USA). The normality was checked by a Shapiro–Wilk’s test (*p* < 0.05). The data were analyzed with one-way ANOVA, followed by Tukey’s test. When the data did not present a normal distribution, the significance was estimated using the Kruskal–Wallis test; in these cases, the nonparametric test was indicated in the pertinent figure legends. The MWM acquisition test data were examined by two-way repeated measures ANOVA. In all cases, the significance level was set at *p* < 0.05. 

## 3. Results

### 3.1. Mice Model NOX5 Expression Characterization

**Figure 1:**
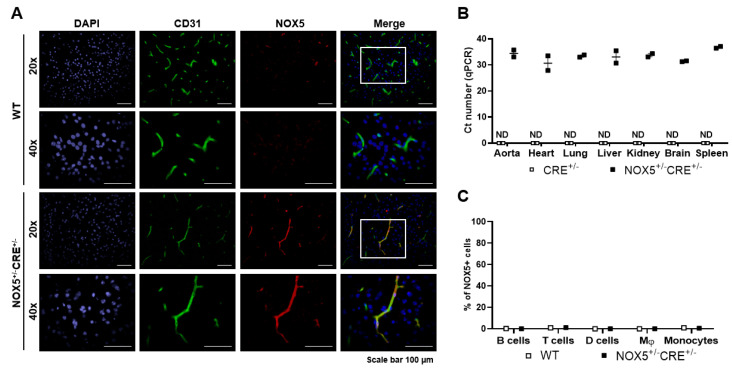
NOX5 expression in organs and circulating cells. (**A**) Representative IF images obtained from the brains of NOX5-expressing mice (*n* = 2; NOX5^+/−^CRE^+/−^) and the negative control (*n* = 1; WT). (**B**) NOX5 mRNA levels in different organs of NOX5-expressing mice (NOX5^+/−^CRE^+/−^) and the negative controls (CRE^+/−^). *n* = 2. ND: nondetected. (**C**) Representative percentage of NOX5 detection by flow cytometry in different subpopulations of circulating cells in the peripheral blood samples. NOX5-expressing mice (*n* = 2; NOX5^+/−^CRE^+/−^) and the negative control (*n* = 1; WT). D cells: dendritic cells and Mφ: macrophages. The results are expressed as individual values ± SEM.

Endothelial-specific NOX5 expression by the *Cdh5*-promoter induction of the CRE recombinase was analyzed in young mice by IF, real-time PCR and by flow cytometry (Figure 1). NOX5 protein expression was detected by IF in the brain of our knock-in mice, and it colocalized with the endothelial CD31 marker (Figure 1A). As expected, a mRNA expression of NOX5 was found in NOX5^+/−^CRE^+/−^ mice organs, whereas no expression was detected in the control mice (CRE^+/−^) (Figure 1B). This is due to the irrigation of these organs and the consequent presence of endothelial cells. In the case of the flow cytometry study, NOX5 expression in the peripheral blood cells was evaluated due to the possible *Cdh5* promoter activation in hematopoietic stem cells. We found no expression of NOX5 proteins either in the myeloid (dendritic cells, monocytes and macrophages) or lymphoid (B cells and T cells) subpopulations (Figure 1C). The NOX5-β-infected TeloHAEC lysates were also analyzed by flow cytometry and used as a positive control. The flow cytometry gating strategy is shown in Figure S2. 

### 3.2. Aging Mice Model Characterization

**Figure 2:**
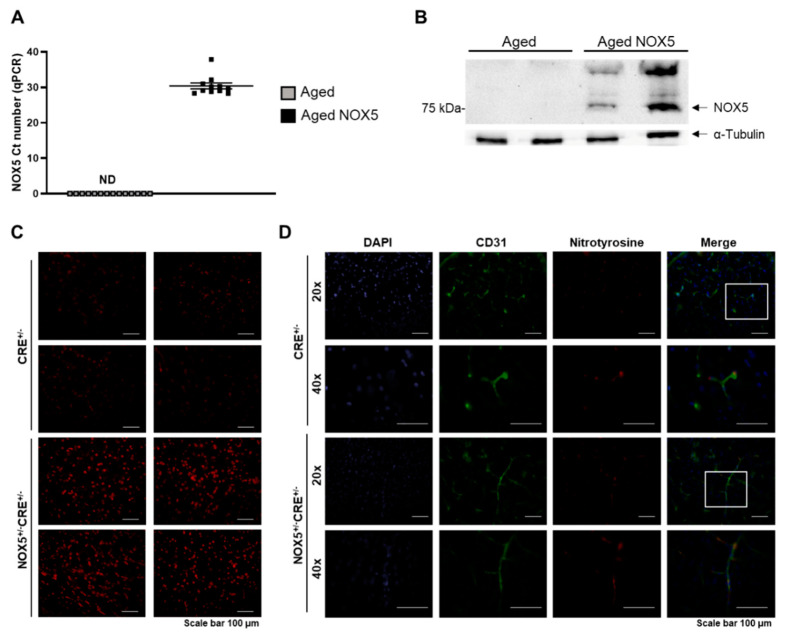
Characterization of the NOX5 knock-in mouse model. (**A**) NOX5 mRNA levels in the brain of aging mice expressing (*Aged NOX5*) NOX5 in the endothelium *vs*. the control mice (*Aged*). *n* = 11–14. ND: nondetected. (**B**) Representative NOX5 and α-tubulin immunoblots in the lungs of aging mice expressing NOX5 in the endothelium (*Aged NOX5*) *vs*. the control mice (*Aged*). (**C**) Intracellular superoxide anion production in young mice. Representative images (20×) obtained from the brain of young mice (2 months old) expressing NOX5 (NOX5^+/−^CRE^+/−^) and their controls (CRE^+/−^). (**D**) Nitrotyrosine accumulation in the young animals. Representative images obtained from the brains of young mice (2 months old) expressing NOX5 (NOX5^+/−^CRE^+/−^) and their controls (CRE^+/−^). The results are expressed as the mean ± SEM. *Aged*: CRE^+/−^ aging mice and *Aged NOX5*: NOX5^+/−^CRE^+/−^ aging mice.

NOX5 protein and mRNA expression was evaluated to analyze whether the conditional knock-in mice remained activated over time (Figure 2). As expected, no mRNA expression of NOX5 was found in the control mice expressing the endothelial-specific CRE recombinase (*Aged*), whereas we detected NOX5 mRNA in our knock-in mice expressing NOX5 (*Aged NOX5*) (Figure 2A). The same occurred when the protein levels were analyzed (Figure 2B). 

Superoxide production, determined by DHE staining, was evaluated in the brain sections to analyze if the conditional knock-in mice generated a functional NOX5 protein. Already, at a young age of 2 months, the NOX5-expressing mice (NOX5^+/−^CRE^+/−^) exhibited higher levels of superoxide than the control mice (CRE^+/−^) (Figure 2C). Moreover, the nitrotyrosine levels, a classical marker of oxidative stress, were also increased in the young NOX5-expressing mice (Figure 2D). As expected, in the aging animals, the nitrotyrosine levels remained enhanced in the brains of the aged NOX5-expressing mice (*Aged NOX5*) compared with the aged control mice (*Aged*) (Figure S3).

**Figure 3:**
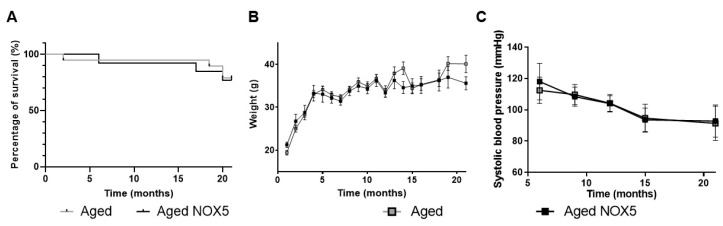
Phenotypic characterization of the aging knock-in mouse model. (**A**) Percent of survival in the aging mice (*Aged* and *Aged NOX5*). *n* = 13–19. (**B**) Mice weight (g) evolution in the *Aged* and *Aged NOX5* groups. *n* = 13–19. (**C**) Systolic blood pressure (mmHg) alterations in the *Aged* and *Aged NOX5* groups. *n* = 13–19. The results are expressed as the mean ± SEM. *Aged*: CRE^+/−^ aging mice and *Aged NOX5*: NOX5^+/−^CRE^+/−^ aging mice.

Additionally, the general parameters, such as survival, weight and blood pressure, were analyzed to evaluate the effects produced by the NOX5 presence in the knock-in mice (Figure 3). No differences were found between the *Aged NOX5* and the *Aged* group in the survival, weight and systolic blood pressure measurements (Figure 3A–C).

**Figure 4:**
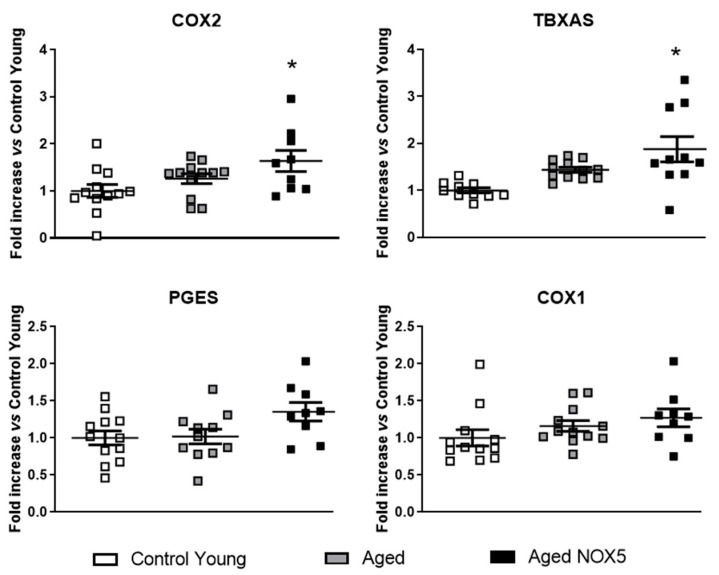
Alterations in mRNA expression of the PG pathway components due to aging and NOX5. The mice cerebral mRNA levels of COX2, TXA2S, PGES and COX1. * *p* < 0.05 *vs*. *Control Young*. *n* = 11–14. The results are expressed as the mean ± SEM. The Kruskal–Wallis test was used for the COX2 analysis, and one-way ANOVA was used for the other genes analyzed. *Control Young*: Young mice, *Aged*: CRE^+/−^ aging mice and *Aged NOX5*: NOX5^+/−^CRE^+/−^ aging mice.

The components of the redox and prostaglandin (PG) pathways were studied in the brains of these mice. NOX2 expression was upregulated in the aging mice compared to the *Control Young*. No significant differences were found between the groups in the NOX4, p22phox, catalase, eNOS and SOD2 mRNA levels (Figure S4). In the case of the PG pathway, COX2 and TXA2S were upregulated in the *Aged NOX5* group compared with *Control Young*, wherease no significant differences were found between the *Control Young* and *Aged* mice (Figure 4).

### 3.3. Behavioral Studies

The *Control Young* mice and Aged mice (*Aged* and *Aged NOX5*) were tested for locomotor activity to assess whether the chronic NOX5 expression in the knock-in mice would have an impact on the mobility of the mice. On the one hand, no differences were found in locomotor activity due to NOX5 expression between the *Aged* and *Aged NOX5* groups (Figure S5). However, aging induced locomotor alterations in the aging mice (*Aged* or *Aged NOX5*) compared to the *Control Young* group, depending on the test used (Figure S5).

**Figure 5:**
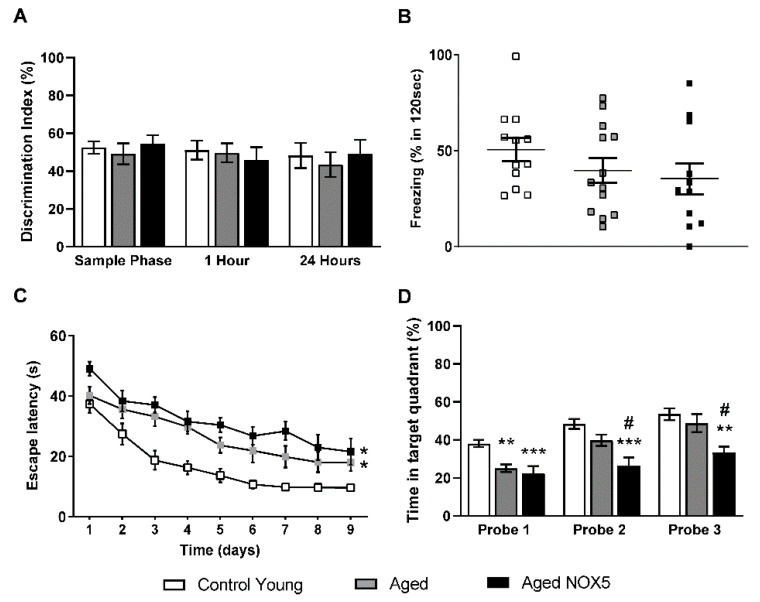
Behavioral tests. (**A**) The Novel Object Recognition Task (NORT) results expressed as the percentage of time spent exploring the new object with respect to the total exploration time (discrimination index). (**B**) The fear conditioning test results expressed as the percentage of time that the mice froze in 120 seconds after the exposition to a new object. (**C**) The Morris water maze (MWM) acquisition phase results are expressed as the time taken to reach the platform (the escape latency) in seconds. * *p* < 0.05 *vs*. *Control Young*. (**D**) The MWM retention phase results are expressed as the percentage of time spent in the quadrant where the platform was previously located. ** *p* < 0.01, *** *p* < 0.001 *vs*. *Control Young* and ^#^ *p* < 0.05 *vs*. *Aged*. *n* = 12–14. The results are expressed as the mean ± SEM. *Control Young*: young mice, *Aged*: CRE^+/−^ aging mice and *Aged NOX5*: NOX5^+/−^CRE^+/−^ aging mice.

On the other hand, NOX5 endothelial expression induced no significant differences in the NORT analysis and the fear conditioning test (Figure 5A,B). In the case of the MWM acquisition phase, the elderly groups, both *Aged* and *Aged NOX5*, showed cognitive deficit, because their escape latencies were significantly higher compared to the Control Young group (Figure 5C). In the MWM retention phase, in probe 2 and probe 3 (at days 7 and 10, respectively), *Aged NOX5* spent a significant shorter time in the correct quadrant compared to the control groups for both *Control Young* and *Aged* (Figure 5D), indicating the presence of memory deficits.

### 3.4. Integrity of the BBB

**Figure 6:**
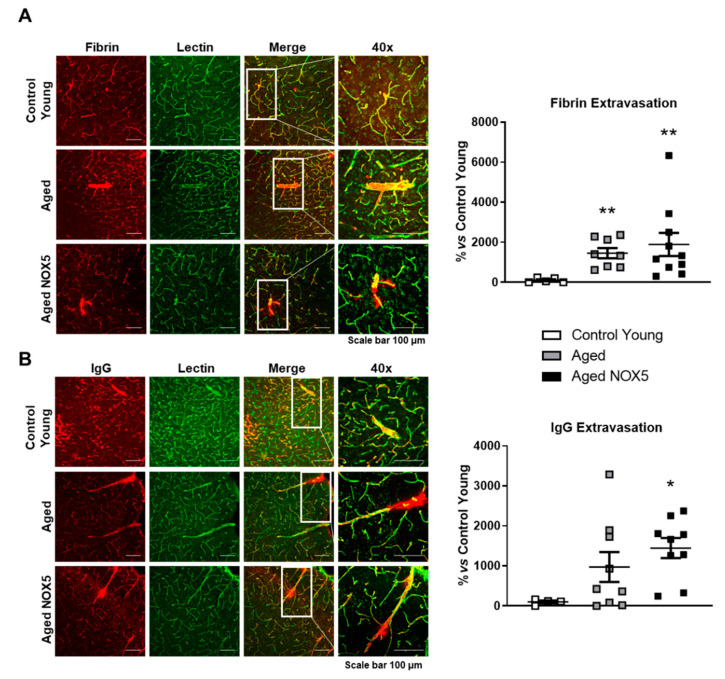
BBB leakage determination by IF assays. A representative confocal microscopy analysis of (**A**) fibrin (red) or (**B**) immunoglobulin G (red) and lectin-positive capillaries (green) on cortical brain sections. * *p* < 0.05 and ** *p* < 0.01 vs. *Control Young*. Scale bar, 100 μm. *n* = 6–10. The results are expressed as the mean ± SEM. Kruskal–Wallis test. *Control Young*: young mice, *Aged*: CRE^+/−^ aging mice and *Aged NOX5*: NOX5^+/−^CRE^+/−^ aging mice.

The BBB integrity was evaluated by the IF staining of fibrin and IgG in the right hemispheres of the *Control Young* mice and aging mice groups (*Aged* and *Aged NOX5*) (Figure 6). Fibrin extravasation was increased in the aging groups (*Aged* and *Aged NOX5*) compared to the *Control Young* group (Figure 6A). In the case of the IgG extravascular levels, only *Aged NOX5* reached a significative difference compared to the *Control Young* (Figure 6B).

### 3.5. Tight Junction Components Expression

**Figure 7:**
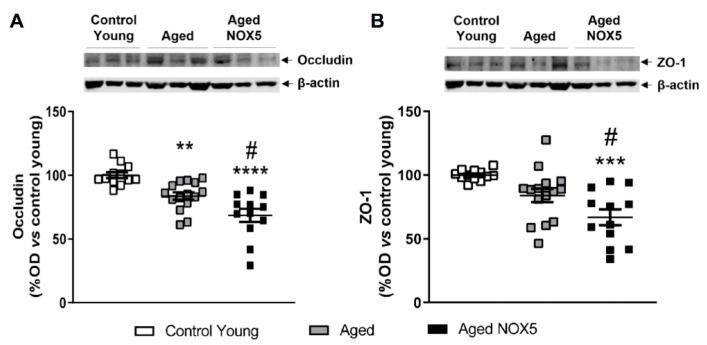
Cerebral occludin and ZO-1 protein expression. Occludin, ZO-1 and β-actin representative immunoblots from mice brain tissue and their quantification. (**A**) Cerebral occludin protein levels expressed as a percentage of the optical density (% OD) in the aging mice *vs*. the *Control Young* group. ** *p* < 0.01, **** *p* < 0.0001 *vs*. *Control Young* and ^#^ *p* < 0.05 *vs*. *Aged*. (**B**) Cerebral ZO-1 protein levels expressed as a percentage of the optical density (% OD) in the aging mice *vs*. the *Control Young* group. ***p < 0.001 *vs*. *Control Young* and ^#^ *p* < 0.05 *vs*. *Aged*. *n* = 13–19. The results are expressed as the mean ± SEM. *Control Young*: young mice, *Aged*: CRE^+/−^ aging mice and *Aged NOX5*: NOX5^+/−^CRE^+/−^ aging mice.

The mRNA and protein levels of two of the main components of the TJ (occludin and ZO-1) were evaluated. No differences were found between the groups when the mRNA expression was analyzed in both of the two genes studied (Figure S6). In the case of the occludin protein expression, a decrease in the aging mice compared to the *Control Young* was found. Additionally, lower protein levels were found in the aging mice expressing NOX5 (*Aged NOX5*) (Figure 7A). The ZO-1 protein levels were also decreased in the *Aged NOX5* group compared to the control groups (*Control Young* and *Aged*) (Figure 7B).

## 4. Discussion

The present work demonstrates a relation between chronic NOX5 endothelial expression, memory loss and TJ components expression alterations, supporting a potential key role in cerebrovascular pathophysiology. Specifically, the main findings obtained in this study were the following: (i) the NOX5 expression in our knock-in mice was limited to the endothelium, and it was maintained a long time; (ii) chronic NOX5 expression induced no changes in the mice survival, weight, blood pressure levels or locomotor activity, but it led to decreased memory in the knock-in aging mice; (iii) chronic NOX5 expression upregulated the mRNA levels of COX2 and TXA2S, both components of the inflammatory PG pathway; (iv) NOX5 expression induced cognitive deficits in aging and (v) chronic NOX5 expression triggered changes in the ZO-1 and occludin protein expression (both components of the TJ) and IgG extravasation, suggesting BBB disruption. 

Oxidative stress constitutes a potential underlying mechanism in the onset and progression of neurodegenerative disorders such as Parkinson’s [29] and Alzheimer’s disease [30]. The brain is known to be not only a potent oxygen consumer but, also, a ROS factory. Additionally, cerebral ROS accumulation and oxidative stress have been shown to increase with aging and to participate in the apparition and progression of neurodegenerative disease [31]. Therefore, neurodegeneration is enhanced by ROS accumulation, including hydrogen peroxide and superoxide anion [32]. 

Many members of the NOX family (NOX2, NOX4 and NOX5) are known to be expressed within the CNS. ROS produced by these enzymes carry out an important role in the neuroinflammatory process and in the pathogenesis of several neurodegenerative diseases [13,14,15,20]. It is postulated that cerebral NOX chronic activation is harmful, specifically NOX2 and NOX4 [16]. In this regard, NOX2 has been associated with aging-related neurodegeneration [33]. Accordingly, in the present study, we found a significant increase in the cerebral NOX2 mRNA levels in the aging mice groups (*Aged* and *Aged NOX5*) comparing to the young mice. 

Furthermore, NOX5 is widely expressed and active among the vasculature [21], and it is known to be a professional producer of superoxide anion [34]. Although NOX5 has been associated with several chronic and acute pathologies, its relevance in the neuropathology remains unclear. For instance, NOX5-dependent ROS increased the systolic blood pressure levels in a podocyte-specific NOX5 expression knock-in mouse model [35] or participated in diabetic nephropathy progression [36]. Moreover, NOX5 is known to potentiate cardiac remodeling and the dysfunction of hypertensive angiotensin II-induced mice expressing NOX5 in the heart under the α-myosin heavy-chain promoter [37]. Additionally, in another knock-in mouse, which expressed NOX5 under the *Tie2* promoter specific to the endothelium and white blood cells, the authors found that chronic NOX5 expression induced elevated systolic blood pressure levels in aging mice [38]. In our study, no differences in the systolic blood pressure measurements in the aging mice were observed. Given our knock-in mice expressed NOX5 under the *Cdh5* promoter, which is specific to the endothelial cells, we could presume that chronic ROS production in the vasculature was not able to have an influence on the hemodynamic status.

NOX5 upregulation and activation is related with many different processes, including the unfolded protein response [39], the management of lipid accumulation and insulin sensitivity [40] or the inflammation via COX2 activation and PG pathway induction [22]. In this context, the COX2 pathway may lead to brain ROS accumulation during aging [41] or after stroke [42]. In this work, we found that cerebral NOX5 expression in our knock-in mice led to an increase in the COX2 mRNA levels compared to the *Control Young* group. Interestingly, we also previously described an upregulation of cardiac COX2 expression in our knock-in mice at the baseline, an effect that was enhanced in the mice suffering from acute myocardial infarction [22]. These data suggest a crosstalk between NOX5 and COX2 under a pathological context. In addition, the TXA2S levels were higher in the *Aged NOX5* than in the *Control Young* mice. TXA2S is a component of the PG pathway that gets active in inflamed and damaged tissues [43]. In addition, TXA2S is known to be a potent vasoconstrictor in brain vessels, and its inhibition has been associated with cerebral blood flow improvement [44]. These data allow us to suggest that NOX5 may have a relevant role in neuroinflammation processes via PG pathway upregulation.

On the other hand, the potential relationship between vascular NOX5 expression and behavior alterations still remains unknown. Interestingly, a specific inhibition of NOX2 prevented the mice from suffering BBB disruption and behavioral changes after a traumatic brain injury [45]. In the present study, we showed that chronic NOX5 endothelial expression induced no locomotor alterations. However, although there are other factors related to cognitive decline associated with aging as the expansion of glia cells, loss of neurons and attenuated vessel density [46], endothelial NOX5-dependent oxidative stress induced memory loss in the retention phase of the MWM in the knock-in aged mice. Taking these data into account, we demonstrated that NOX5 expression in the brain vasculature alters the cognitive status of aging NOX5-expressing mice.

Vascular dysfunction in the brain, and, therefore, BBB disruption, is a strongly demonstrated mechanism of neurodegenerative disease apparition and progression [47,48]. Recently, Casas et al. described that endothelial NOX5-mediated ROS in their knock-in mice enhanced the poststroke BBB impairment and infarct size [20]. Alterations in the BBB integrity can lead to several pathologic events, such as the leakage of plasma proteins, the infiltration of immune cells, the activation of microglial cells and localized and increased cytokine production. These could be associated with the increased cerebral infarct size in the NOX5-expressing mice, performed by Casas et al. [49]. Therefore, NOX5 may participate in neuronal degeneration mediated by BBB alterations triggered via ROS accumulation that allow to postulate this oxidase as a therapeutic target in cerebral ischemic injury. In our study, these BBB alterations due to NOX5 may not be mediated by increased systolic blood pressure levels [38] but through other mechanisms, like COX2 activation. To analyze the BBB integrity, we evaluated the cerebral extravasation of fibrin and IgG, and we found a higher tendency of fibrin extravasation in the knock-in mice. In the case of the extravascular IgG levels, we obtained a significant increase in the *Aged NOX5*. This increase of BBB permeability could be due to alterations in the TJ, as it is known that TJ are responsible for the BBB function as barrier [6]. We found no mRNA alterations of occludin and ZO-1, two components of TJ, but their protein levels decreased in the aging mice due to NOX5 endothelial expression. These findings suggest that NOX5 could be triggering occludin and ZO-1 degradation or preventing these proteins from being produced by the endothelial cells. Occludin is a key transmembrane regulator of BBB functional integrity, and ZO-1 is crucial for the stability and function of TJ [19]. Collectively, this supports that NOX5 weakens the TJ integrity and, subsequently, the BBB.

## 5. Conclusions

In summary, we demonstrated for the first time that the endothelial chronic expression of NOX5 induces changes not only in the inflammatory PG pathway and TJ structure (occludin and ZO-1 expression) but, also, in the memory loss of humanized NOX5-expressing aging mice. Given that NOX5 expression is related to BBB breakdown [20], our findings support the idea that NOX5 may play a relevant role in BBB alterations during aging. In this context, we consider NOX5 as a new therapeutic target, as its inactivation could prevent the BBB disruption associated with aging.

## Figures and Tables

**Table 1:** The primers used for cDNA amplification.

Gene	Accession Number		Primers
*NOX2*	NM_007807.5	forward	5’-ACTCCTTGGGTCAGCACTGG-3’
		reverse	5’-GTTCCTGTCCAGTTGTCTTCG-3’
*NOX4*	NM_001285833.1	forward	5’-GGAGACTGGACAGAACGATTCC-3’
		reverse	5’-TGTATAACTTAGGGTAA TTTCTAGAGTGAATGA-3’
*NOX5*	NM_001184780.2	forward	5’-ATGAGTGCCGAGGAGGATG-3’
		reverse	5’-ATCGATGGCAGTGGCTCCAT-3’
*CATALASE*	NM_009804.2	forward	5’-GCTGAGAAGCCTAAGAACGCAAT-3’
		reverse	5’-CCCTTCGCAGCCATGTG-3’
*eNOS*	NM_008713.4	forward	5’-CTGGAGCACCCCACGCT-3’
		reverse	5’-AGCGGTGAGGGTCACACAG-3’
*p22phox*	NM_007806	forward	5’-GCCCTCCACTTCCTGTT-3’
		reverse	5’-GCAGATAGATCACACTGGCAAT-3’
*SOD2*	NM_013671	forward	5’-CACACATTAACGCGCAGATCA-3’
		reverse	5’-GGTGGCGTTGAGATTGTTCA-3’
*COX1*	NM_008969.4	forward	5’-ACTCACAGTGCGGTCCAAC-3’
		reverse	5’-AACTCCCTTCTCAGCAGCAG-3’
*COX2*	NM_011198.4	forward	5’-TTCGGGAGCACAACAGAGT-3’
		reverse	5’-TAACCGCTCAGGTGTTGCAC-3’
*PGES*	NM_022415.3	forward	5’-AGGATGCGCTGAAACGTGGAG-3’
		reverse	5’-CCGAGGAAGAGGAAAGGATAG-3’
*TXA2S*	NM_011539.3	forward	5’-AACAGAATGGCCTCAGGTCT-3’
		reverse	5’-AGTTCACAGGCTTGGCTGAT-3’
*OCCLUDIN*	NM_001360536.1	forward	5’-CGGCAGGTTCGCTTATCTT-3’
		reverse	5’-TGTCATTGCTTGGTGCATAA-3’
*ZO-1*	NM_009386.2	forward	5’-CCGCTAAGAGCACAGCAAT-3’
		reverse	5’-CATTGCAACTCGGTCATTTT-3’
*GAPDH*	NM_008084.3	forward	5’-ATGACAACTTTGTCAAGCTCATTT-3’
		reverse	5’-GGTCCACCACCCTGTTGCT-3’

Endothelial nitric oxide synthase (eNOS), Superoxide dismutase 2 (SOD2), Cyclooxygenase 1 (COX1), Cyclooxygenase 2 (COX2), Prostaglandin E synthase (PGES) and Thromboxane A2 synthase (TXA2S).

**Table 2:** Monoclonal antibodies used to differentiate the blood subpopulations in the mice samples.

Protein	Fluorophore	Brand	Clone	Dilution
CD11b	BV-510	Biolegend	M1/70	1:100
B220	BV-395	BD Biosciences	RA3-6B2	1:100
CD3	PE/Cy7	Biolegend	17A2	1:100
CD11c	PE	Biolegend	N418	1:100
Ly-6C	BV-605	Biolegend	HK1.4	1:100
F4/80	APC/Cy7	Biolegend	BM8	1:100

## Data Availability

Data is contained within the article.

## Supplementary Materials

The following are available online at https://www.mdpi.com/article/10.3390/antiox10081311/s1: Figure S1: Generation of an endothelial CRE-expressing knock-in mouse. Figure S2: NOX5 detection by flow cytometry. Figure S3: Nitrotyrosine immunofluorescence of aging mice. Figure S4: Alterations in mRNA expression of the redox pathway components due to aging and NOX5. Figure S5: Locomotor activity tests. Figure S6: Occludin and ZO-1 mRNA expression.
