# Supplementary material for: Expression of Endothelial NOX5 Alters the Integrity of the Blood-Brain Barrier and Causes Loss of Memory in Aging Mice
Source: Antioxidants (Basel). 2021 Aug 20;10(8):1311. doi: 10.3390/antiox10081311 (PMC8389305; doi:10.3390/antiox10081311)
Supplement: Supplementary file 1 [file antioxidants-10-01311-s001.zip › antioxidants-1325519-supplementary.pdf]

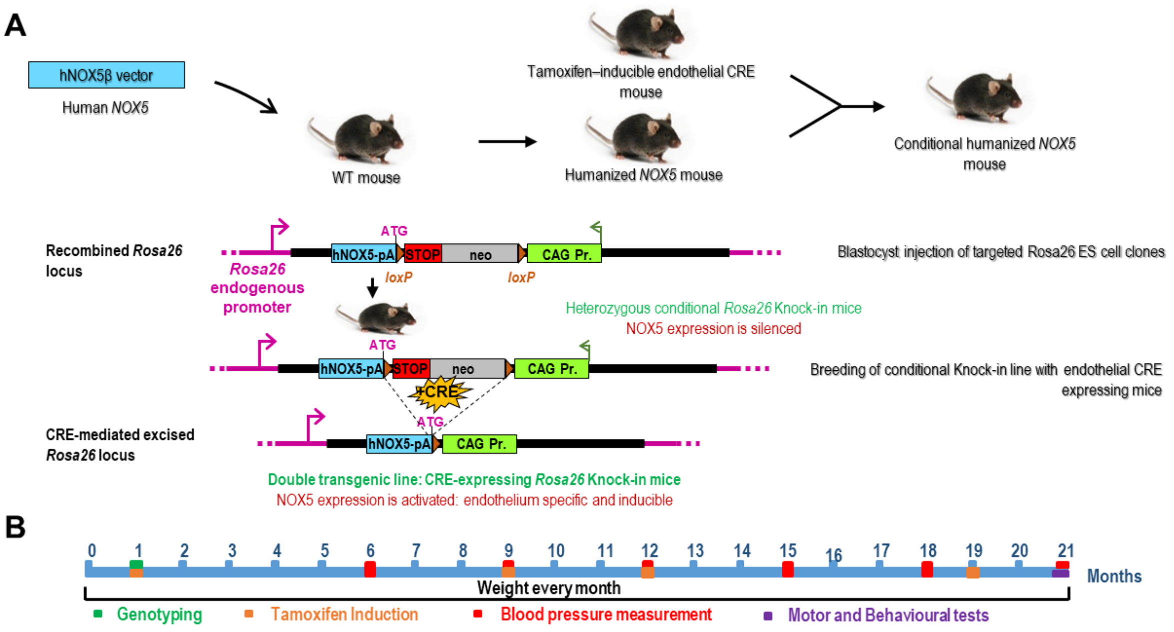

Figure S1. Generation of an endothelial CRE-expressing knock-in mouse. (**A**) Representative schematic of a humanized NOX5 mouse. NOX5 gene located on chromosome 6 was engineered in heterozygosis, thus resulting in mice with a single copy of the gene in both males and females. Construction of a conditional humanized NOX5 knock-in mouse. Purple and black lines represent genomic sequences located in the endogenous Rosa26 locus and in the targeting vector to insert NOX5, respectively. LoxP sites are shown as brown triangles. The NOX5 cDNA plus hGH polyA are depicted as a blue box. The combined STOP-neomycin cassette is represented by a red-grey box and the CAG promoter by a green box. (**B**) Representative schedule of humanized NOX5 aging mice.

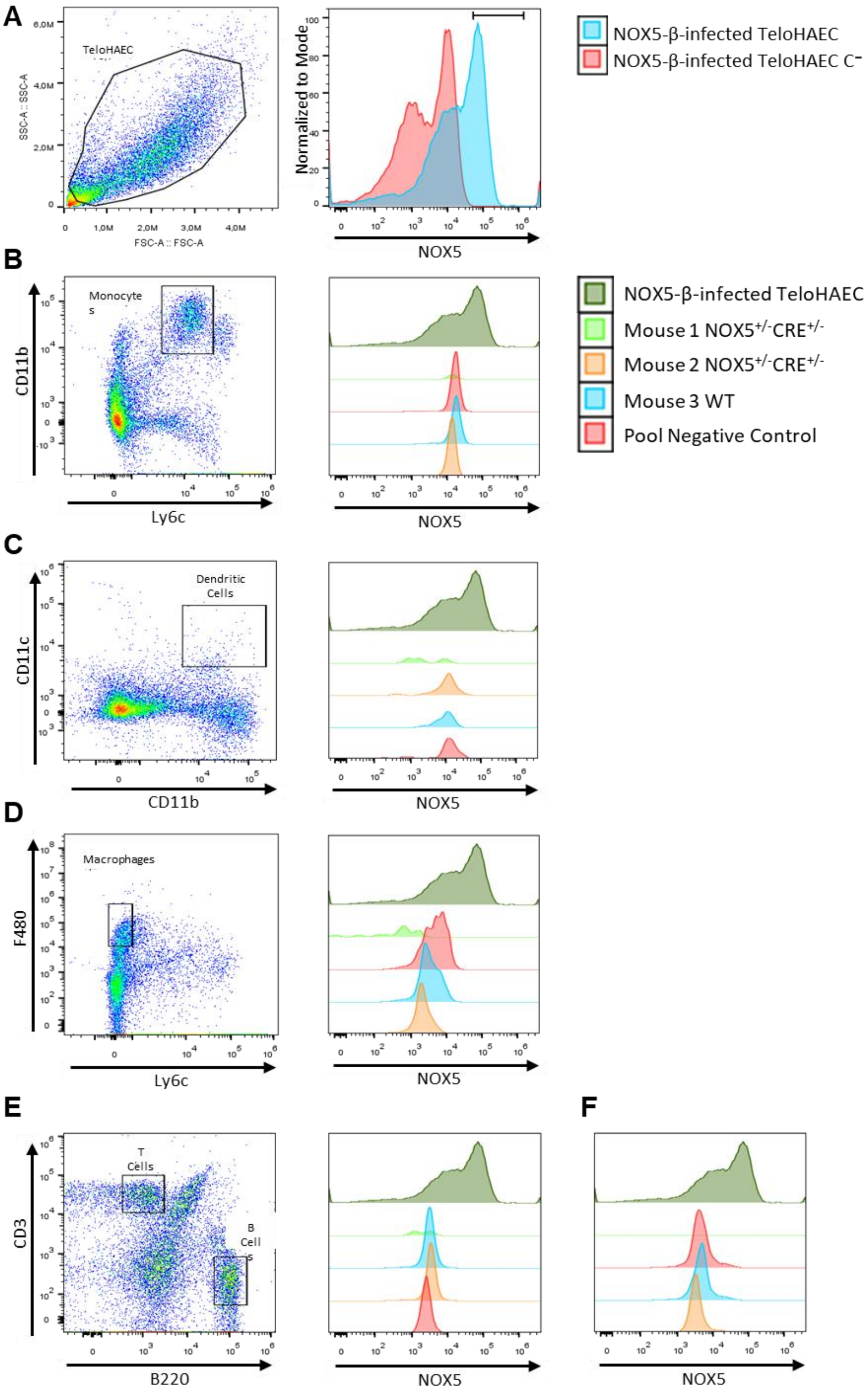

Figure S2. NOX5 detection by flow cytometry. (**A**) Gating strategy and NOX5 histogram of NOX5-β-infected TeloHAEC used as a positive control for NOX5 detection by flow cytometry. (**B**) Gating strategy to identify monocytes and the NOX5 histogram of NOX5$^{+/-}$CRE$^{+/-}$ or WT mice in addition to the positive control in the monocytes. (**C**) Gating strategy to identify dendritic cells and the NOX5 histogram of NOX5$^{+/-}$CRE$^{+/-}$ or WT mice in addition to the positive control in dendritic cells. (**D**) Gating strategy to identify macrophages and the NOX5 histogram of NOX5$^{+/-}$CRE$^{+/-}$ or WT mice in addition to the positive control in macrophages. (**E**) Gating strategy to identify B and T cells and the NOX5 histogram of NOX5$^{+/-}$CRE$^{+/-}$ or WT mice in addition to the positive control in B cells. (**F**) The NOX5 histogram of NOX5$^{+/-}$CRE$^{+/-}$ or WT mice in addition to the positive control in T cells.

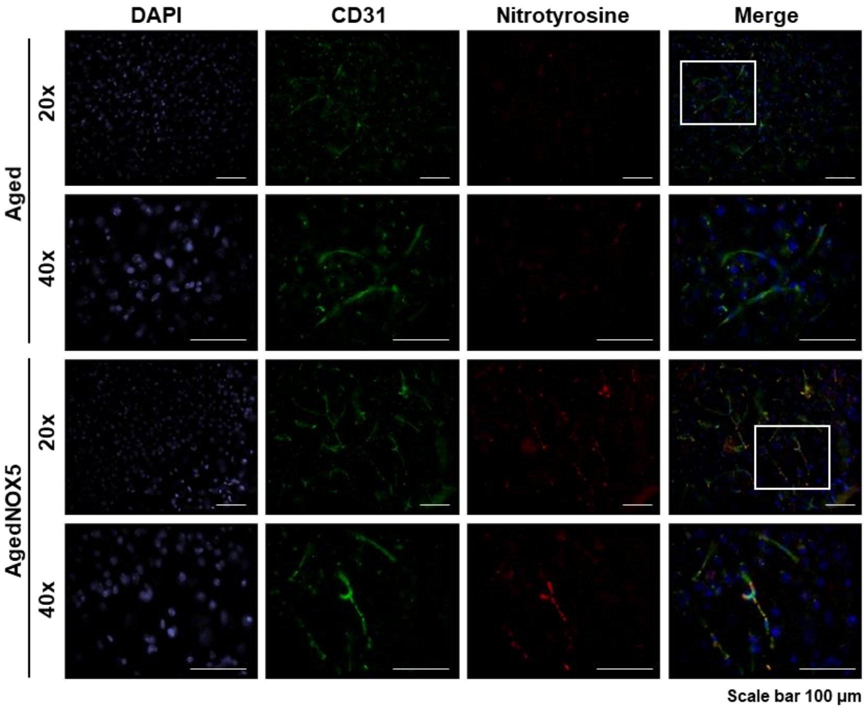

Figure S3. Nitrotyrosine immunofluorescence of aging mice. Representative IF images obtained from the brains of the aging mice (*Aged* and *AgedNOX5*).

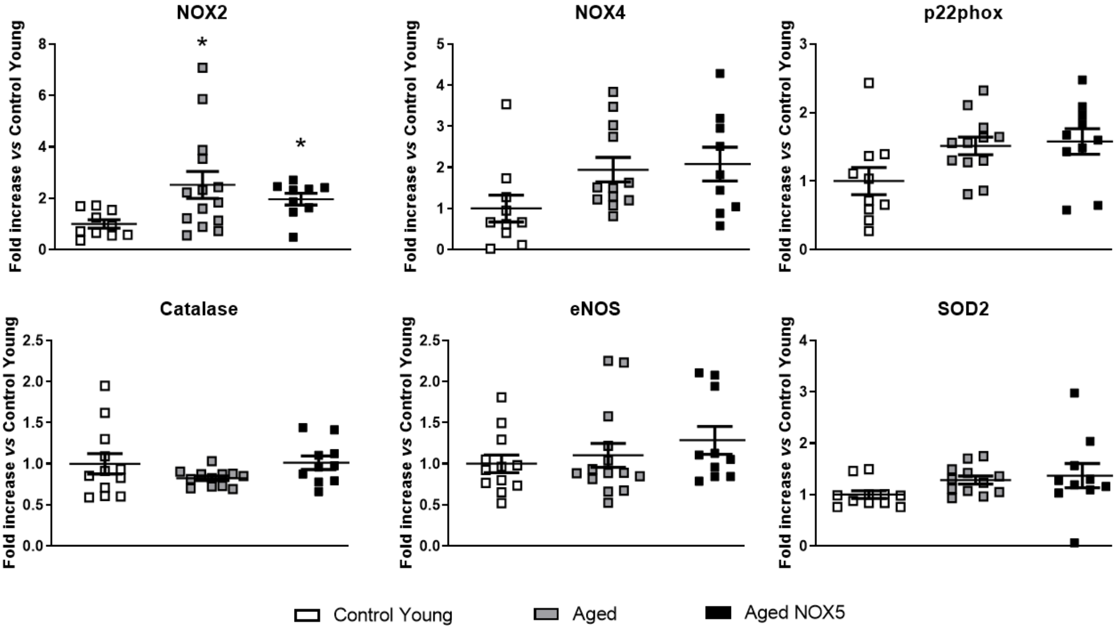

Figure S4. Alterations in mRNA expression of the redox pathway components due to aging and NOX5. Mice cerebral mRNA levels of the expression of NOX2, NOX4, p22phox, catalase, eNOS and SOD2, all components of the redox-signaling pathway. *$p < 0.05$ vs. *Control Young*. $n = 11–14$. Results are expressed as the mean ± SEM. *Control Young*: young mice, *Aged*: $CRE^{+/-}$ aging mice and *Aged NOX5*: $NOX5^{+/-}CRE^{+/-}$ aging mice.

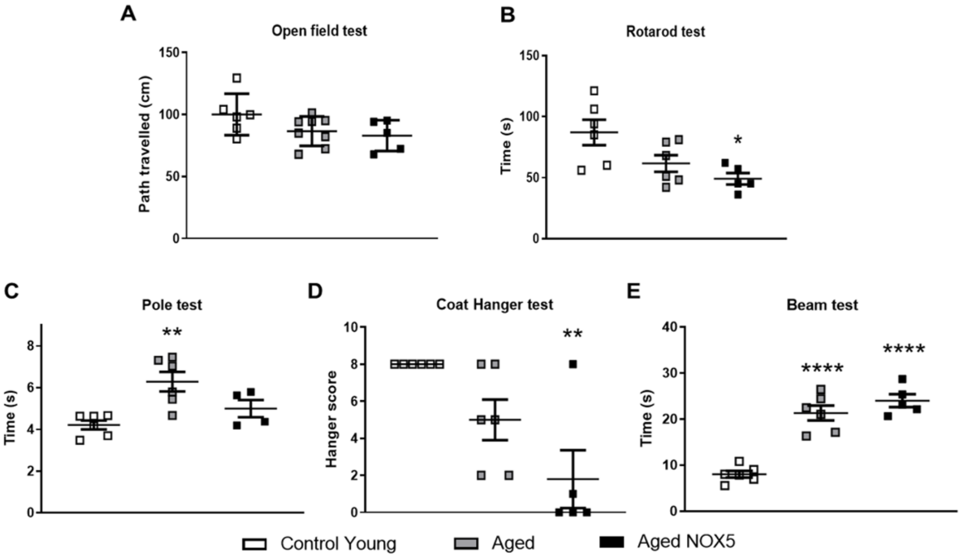

Figure S5. Locomotor activity tests. (**A**) Open field study results are expressed as centimeters (cm) of the distance traveled. (**B**) The Rotarod results are expressed as the average time of the latency to fall (s). $*p < 0.05$ *vs*. *Control Young*. (**C**) The Pole test results are expressed as the average time spent by mice in turning downward and descending the pole (s). $**p < 0.01$ vs. *Control Young*. (**D**) The Coat Hanger test results are expressed as the average scores given to the mice. $**p < 0.01$ vs. *Control Young*. Kruskal–Wallis. (**E**) The Beam test results are expressed as the average time taken to cross the beam to the safe cage (s). $****p < 0.001$ vs. *Control Young*. $n = 6–8$. The results are expressed as the mean ± SEM. *Control Young*: young mice, *Aged*: $CRE^{+/-}$ aging mice and *Aged NOX5*: $NOX5^{+/-}$ $CRE^{+/-}$ aging mice.

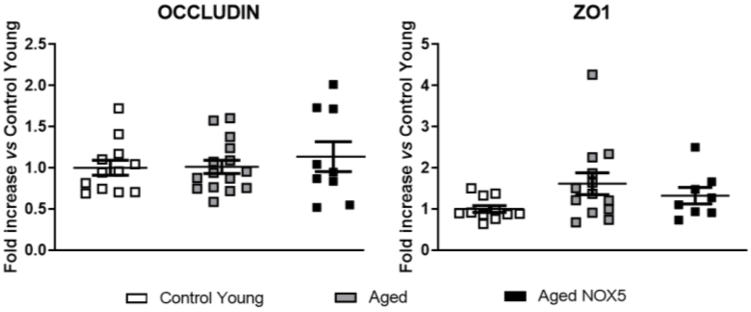

Figure S6. Occludin and ZO-1 mRNA expression. Cerebral occluding and ZO-1 mRNA expression in the aging mice vs. the *Control Young* group. $n = 11–15$. The results are expressed as the mean ± SEM. *Control Young*: young mice, *Aged*: $CRE^{+/-}$ aging mice and *Aged NOX5*: $NOX5^{+/-}CRE^{+/-}$ aging mice.
